# Bone marrow mesenchymal stem cell-secreted exosomes carrying microRNA-125b protect against myocardial ischemia reperfusion injury via targeting SIRT7

**DOI:** 10.1007/s11010-019-03671-z

**Published:** 2019-12-19

**Authors:** Qi Chen, Yu Liu, Xueyan Ding, Qinfeng Li, Fuyu Qiu, Meihui Wang, Zhida Shen, Hao Zheng, Guosheng Fu

**Affiliations:** 1 Department of Cardiology, School of Medicine, Sir Run Run Shaw Hospital, Biomedical Research Center, Zhejiang University, No. 3, East Qingchun Road, Hangzhou, 310016 Zhejiang China; 2 Department of Cardiology, Nanjing University Medical School Affiliated Nanjing Drum Tower Hospital, No. 321, Zhongshan Road, Nanjing, 210008 Jiangsu China; 3 Department of Cardiology, Zhejiang Provincial People’s Hospital, No. 158, Shangtang Road, Hangzhou, 310014 Zhejiang China

**Keywords:** Ischemia reperfusion, Exosomes, miR-125b, Apoptosis, Inflammatory factor

## Abstract

MicroRNA-125b (miR-125b) reduces myocardial infarct area and restrains myocardial ischemia reperfusion injury (I/R). In this study, we aimed to investigate the effect of bone marrow mesenchymal stem cell (BMSC)-derived exosomes carrying miR-125b on I/R rats. The myocardial I/R model in rats was constructed by ligation of the left anterior descending coronary artery (LAD). Rats were randomly divided into I/R and Sham group. Lv-cel-miR-67 (control) or Lv-miR-125b was transfected into BMSCs. Exosomes were extracted from transfected BMSCs, and separately named BMSC-Exo-67, BMSC-Exo-125b, and BMSC-Exo. MTT assay and flow cytometry were used to detect the viability and apoptosis of I/R myocardium cells, respectively. The expression of cell apoptosis proteins and the levels of inflammatory factors were examined by Western blot and ELISA assay, respectively. The target relationship between miR-125b and SIRT7 was predicted by using StarBase3.0, and was confirmed by using dual-luciferase reporter gene assay. qRT-PCR, immunohistochemistry staining, and Western blot were used to evaluate the expression of SIRT7 in myocardium tissues in I/R rats. BMSC-derived exosomes were successfully isolated and identified by TEM and positive expression of CD9 and CD63. The expression of miR-125b was down-regulated in I/R myocardium tissues and cells. BMSC-Exo-125b significantly up-regulated miR-125b in I/R myocardium cells. The intervention of BMSC-Exo-125b significantly increased the cell viability, decreased the apoptotic ratio, down-regulated Bax and caspase-3, up-regulated Bcl-2, and decreased the levels of IL-1β, IL-6, and TNF-α in I/R myocardium cells. SIRT7 was a target of miR-125b, and BMSC-Exo-125b significantly down-regulated SIRT7 in myocardium cells. In addition, the injection of BMSC-Exo-125b alleviated the pathological damages and down-regulated SIRT7 in myocardium tissues of I/R rats. BMSC-derived exosomes carrying miR-125b protected against myocardial I/R by targeting SIRT7.

## Introduction

Myocardial ischemia reperfusion is clinicopathological criteria defined as insufficiency of blood supply to the heart and subsequent recovery of perfusion combined with reoxygenation [1]. Reperfusion is indispensable for the survival of myocardial ischemia tissues, but an amount of proof indicates that reperfusion itself induces irretrievable additional tissue injury [2]. Myocardial ischemia reperfusion injury (I/R) can induce the apoptosis and necrosis of cardiomyocytes or even cardiac arrest, thereby influencing the treatment outcome of heart diseases [3]. It is urgent to find practical and effective therapeutic methods for I/R.

Bone marrow mesenchymal stem cells (BMSCs) are fibroblast-like, pluripotent adult stem cells [4] that exist in the bone marrow microenvironment. BMSCs reduce intestinal I/R in rats [5]. The injection of MSCs secreted by adult bone marrow into the infarction area can decrease infarct size and repair the function of the heart after I/R [6, 7]. Exosomes, as a type of membrane vesicle, have been viewed as a medium to promote intercellular communication and regulate the recipient cell function through delivering proteins, RNAs, and other molecular constituents [8]. BMSC-derived exosomes protect against testicular I/R owing to the activities of anti-oxidant, anti-inflammatory, and anti-apoptosis [9]. Recently, exosomes derived from BMSCs have been reported to promote cell proliferation and survival through the transportation of microRNAs (miRNAs) [10]. BMSCs prevent against renal I/R by secretion of exosomes loaded with miR-199a-5p that can target BIP to suppress endoplasmic reticulum stress at early stages of reperfusion [11].

MiRNAs are defined as 21–23 nucleotide non-coding RNA molecules, and regarded as novel regulatory factors of gene expression via binding to target messenger at post-transcriptional level [12–14]. Numerous studies have revealed that miRNAs modulate the expression of critical proteins involved in I/R [15]. For instance, the expression of miR-15 and miR-15b are increased in myocardial I/R model of mice, and the down-regulation of miR-15a/b may be a prospective strategy to inhibit I/R-induced apoptosis of myocardium cells [16]. Overexpression of miR-125b weakens the apoptosis of myocardium cells induced by myocardial I/R through preventing p53-mediated apoptotic signaling and inhibiting TRAF6-mediated activation of NF-kB [17]. Recently, miRNAs loaded in exosomes have also been reported, demonstrating that exosomes may act as a miRNA transport to regulate intercellular communication [18]. Exosomes derived from mesenchymal stromal cells (MSCs) attenuate myocardial I/R through miR-182-regulated macrophage polarization [19]. However, research involving the effect of BMSC-derived exosomes carrying miR-125 on myocardial I/R remains limited.

In this study, the protective effects of BMSC-derived exosomes carrying miR-125 in myocardial I/R were evaluated. After isolation of BMSC-derived exosomes, the effects of BMSC-Exo-125b on the viability and apoptosis of I/R myocardium cells were detected. Then, we examined the correlation between miR-125b and SIRT7, followed by the effects of BMSC-Exo-125b on cardiac function of I/R rats. Our findings convinced that BMSC-Exo-125b recovered the cardiac function of I/R rats through down-regulation of SIRT7, which might reveal a new therapeutic approach for myocardial I/R.

## Methods

### Isolation and incubation of BMSCs

All animal experimental procedures were permitted by the Ethics Committee of our hospital, and were performed in accordance with the Guide for the Care and Use of Laboratory Animals (eighth edition, 2011, National Institutes of Health, USA). Two male Sprague–Dawley (SD) rats (Shanghai SLAC Laboratory Animal Co., Ltd, Shanghai, China), aged 10–14 days, weighing 60–100 g, were used for experiments. BMSCs were isolated from the femur and tibia of rats. Red blood cells were lysed using ACK lysis buffer (0.15 M NH4Cl, 1 mM KHCO3, 0.1 mM Na2EDTA), washed, resuspended, and cultured in DMEM/F12 (Gibco, Carlsbad, CA, USA) containing 40% MCDB201, 100 U/mL penicillin, 100 U/mL streptomycin, and 2% fetal bovine serum. BMSCs were cultured in an incubator at 37 °C, 5% CO_2_ with saturated humidity. After 48 h of incubation, the medium was changed, and cells were passaged when reaching 80–90% confluence.

### Flow cytometry analysis

When BMSCs at the third-passage reaching 80% confluence, the cells were trypsinized, washed, centrifuged, and resuspended in phosphate-buffered saline (PBS). BMSCs were incubated with antibodies against CD44, CD105, and CD31 (Neomarker, CA, USA), and then analyzed by a flow cytometry (FACSCalibur; BD, Alaska, MN, USA).

### Isolation and characterization of exosomes

Exosomes were extracted from the supernatant of BMSCs using an ExoQuick-TC Kit (Invitrogen, Waltham, MA, USA) according to manufacturer’s instructions. A total of 10 μL exosome pellets were transferred to carbon-coated 200-mesh copper electron microscopy grids and cultured for 1 min at room temperature. Then, exosomes were stained with 3% (w/v) sodium phosphowolframate (pH = 6.8) for 5 min, and washed with double distilled water. After being left to dry at room temperature, exosomes were observed under a transmission electron microscope (Hitachi H7500 TEM, Tokyo, Japan). Micrographs were used to identify exosomes in BMSCs. Twenty exosomes were randomly selected to measure the diameters.

### Establishment of myocardial I/R model in rats

Twenty male SD rats (Shanghai SLAC Laboratory Animal Co., Ltd), weighing 180–200 g, aged 6 weeks, were randomly divided into Sham group and I/R group. Rats were anesthetized by intraperitoneal injection of sodium pentobarbital (50 mg/kg). Ischemia was achieved by ligation of the left anterior descending coronary artery (LAD) using a 6.0 prolene suture for 30 min. Then the knot was relaxed and the heart was allowed reperfusion for 2 h. The same procedure was performed on Sham group without LAD. In order to examine the effect that BMSC-Exo-miR-125b exhibit on I/R, BMSC-Exo-miR-67 (50 µg) or BMSC-Exo-miR-125b (50 µg) was injected into the ligation zone adjacent to the left anterior free wall after left ventricle exposure. Myocardial tissues were collected and used for the subsequent assays.

### Determination of cardiac function and myocardial infarct size

After modeling for 1 week, transthorax echocardiography (TTE) was used to evaluate the cardiac function of I/R rats according to the following parameters: the left ventricular ejection fraction (LVEF), left ventricular fraction shortening (LVFS), left ventricular systolic pressure (LVSP), left ventricular end systolic diameter (LVESD), left ventricular end-diastolic dimension (LVEDD), left ventricular end-diastolic pressure (LVEDP), the left ventricular d*p*/d*t* curve, maximal rate of pressure rise (+ d*p*/d*t*_max_), and decline (− d*p*/d*t*_max_).

The myocardial infarct size was determined by triphenyltetrazolium chloride (TTC) staining. Briefly, the heart of rat was sliced into small sections and were incubated with 1% TTC (A610558, SangonBootech Co, shanghai, China) for 30 min, fixed with 10% formalin for 10 min, and then observed after rinsing. The infarct area (pale white) and the area at risk (brick red) were measured using Image-Pro Plus 6.0 software. The infarct size (%) was calculated as infarct area/area at risk × 100%.

### Hematoxylin–eosin (HE) staining

The myocardium tissues were fixed with 10% formaldehyde for 24 h, and then sliced into 4-µm sections. Subsequently, tissue sections were stained with Hematoxylin and Eosin. The pathological changes of myocardium tissues were observed under microscope.

### Acquirement and incubation of cardiac myocytes in rats

Rats of Sham and I/R groups were injected with sodium pentobarbital (30 mg/kg) for anesthesia, and then the chest was opened and the heart was extracted quickly. The ventricular tissues were then dissected, cut into blocks of about 2 mm^3^, and digested by pancreatin and type II collagenase. Dispersed cells were suspended in DMEM containing with 10% fetal bovine serum (FBS) and 1% penicillin/streptomycin, and then filtered with a 70-μm cell strainer. The isolated cardiomyocytes were cultured in an incubator at 37 °C, 5% CO_2_ with saturated humidity. The cardiac myocytes isolated from Sham and I/R group were named Sham-C and I/R–C, respectively.

### Cell transfection

BMSCs were seeded in 6-well plates at a density of 6 × 10^5^ per well, and cultured in an incubator at 37 °C, 5% CO_2_ overnight. BMSCs were transfected with Lv-cel-miR-67 (control) or Lv-miR-125b (Vigene Biosciences, Rockville, MD, USA) by using Lipofectamine 2000 (Invitrogen) at a multiplicity of infection (MOI) of 10 in the presence of polybrene (8 µg/mL; Sigma-Aldrich, St. Louis, MO, USA) for 24 h according to manufacturer’s recommendations. Cells transfected with Lv-cel-miR-67 (control) or Lv-miR-125b were named BMSC-67 or BMSC-125b. BMSCs without transfection were considered as the Mock group. After 48 h of culturing, exosomes were isolated from the above BMSCs, and separately named BMSC-Exo-67, BMSC-Exo-125b, and BMSC-Exo. Exosomes were identified by the micro-morphology under transmission electron microscope and positive expression of CD9 and CD63. The transfection efficiency was identified by qRT-PCR.

### The treatment of I/R cardiomyocytes with exosomes

Cardiomyocytes were treated with BMSC-derived exosomes (MSC-Exo, MSC-Exo-67, and MSC-Exo-125b) in DMEM medium (Hyclone, South Logan, UT, USA) for 48 h. The intervention efficiency was identified by PKH26 (Sigma-Aldrich)-labeled in exosomes under a fluorescence microscopy (Leica, xsp-63xd, Wetzlar, Germany).

### qRT-PCR

Total RNA was extracted from tissues and cells by using TRIzol™ Plus RNA Isolation Reagents (Invitrogen). The reverse transcription kit (Takara, Otsu, Japan) was applied for RNAs reverse transcription. qRT-PCR was performed on ABI 7500HT Fast Real-Time PCR System (Applied Biosystems, CA, USA) under the following reaction conditions: 95 °C for 3 min, 40 cycles at 95 °C for 15 s, 60 °C for 30 s, and 72 °C for 20 s. The mRNA expression level was calculated using the $2^{{ - \Delta \Delta C_{\text{t}} }}$ method. The primer sequences are shown in Table 1. U6 or β–actin was used as the internal reference of miR-125b or SIRT7, respectively.

**Table 1:** Primer sequences

Name of primer	Sequences
miR-125b	Forward: 5′-GAATCCCTGAGACCCTAAC-3′
	Reverse: 5′-GTGCAGGGTCCGAGGT-3′
U6	Forward: 5′-CTCGCTTCGGCAGCACA-3′ Reverse: 5′-AACGCTTCACGAATTTGCGT-3′
SIRT7	Forward: 5′-TCTCTGAGCTCCATGGGAAT-3′
	Reverse: 5′-CATGAGGAGCCGCATTACAT-3′
β-Actin	Forward: 5′-ACACCTTCTACAATGAGCTG-3′
	Reverse: 5′-CTGCTTGCTGATCCACATCT-3′

### Western blot

Total proteins were extracted using RIPA lysis buffer (Beyotime Biotechnology, Shanghai, China) and then quantified using BCA Protein Assay Kit (ThermoFisher, Shanghai, China). The protein samples were mixed with 5 × loading buffer and separated by 10% sodium dodecyl sulfate (SDS)-polyacrylamide gel electrophoresis. Next, the proteins were transferred to a polyvinylidene fluoride (PVDF) membrane, and immersed in 5.0% non-fat milk for 45 min at 37 °C. The membrane was then incubated with primary antibodies, including β-actin (1:1000, ab179467, Abcam), CD9 (1:1000, ab92726, Abcam), CD81 (1:1000, ab108950, Abcam), Bcl-2 (1:1000, ab196495, Abcam), Bax (1:1000, ab199677, Abcam), caspase-3 (1:1000, ab49822, Abcam), and SIRT7 (1:1000, ab78977, Abcam) at 4 °C overnight. Subsequently, the membrane was incubated with HRP-conjugated goat anti-rabbit IgG (1:10000, Sigma, USA) for 1 h at room temperature. Protein bands were visualized with Chemiluminescent Substrate kit. β-actin was used as the internal reference.

### MTT assay

Myocardium cells were seeded in 96-well plates (6 × 10^3^ cells/well), and BMSC-Exo-125b was added into each well, and incubated for 0, 24, 48, and 72 h, respectively. Subsequently, 20 μL MTT (5 mg/mL, Sigma-Aldrich) was pipetted into each well. After 4 h of incubation, 150 μL DMSO was added for reaction termination. The optical density at 495 nm (OD495) was measured by a microplate reader (Applied Biosystems).

### Annexin V-PI double staining

Cells were stained using an Annexin V-PI kit (Invitrogen). A total of 1 × 10^5^ cells were suspended in 500 µL binding buffer, and then stained with 5 µL Annexin V‐EGFP and 5 mL Propidium Iodide, respectively, at room temperature for 10 min in the dark. The cell apoptosis was analyzed on a MUSE™ flow cytometer (Merck Millipore, USA). Lower left quadrant (LL) represented viable cells; upper left quadrant (UL) represented necrotic cells; lower right quadrant (LR) represented early apoptotic cells; upper right quadrant (UR) represented late apoptotic cells. The apoptotic ratio (%) was calculated as the cell ratio in LR + UR.

### ELISA assay

Cardiomyocytes of each group were centrifuged at 5000×*g* at 4 °C for 10 min, and the supernatant was collected. The levels of IL-1β, IL-6, and TNFa were measured by using OptEIA™ mouse cytokine kits (Thermo Fisher Scientific) according to manufacturer’s instructions.

### Dual-luciferase reporter assay

A binding site at 3′-UTR of SIRT7 was predicted on miR-125bby StarBase3.0. According to the predication, SIRT7-Mut and SIRT7-Wt were cloned and combined with PsiCHECK-2 vector (Promega, Madison, USA). SIRT7-Mut or SIRT7-Wt was co-transfected with miR-125b or miR-NC (GenePharma Co., Ltd, Shanghai, China) into myocardium cells with Lipofectamine 3000 (L3000015, Thermo Fisher Scientific). After 48 h of transfection, the luciferase activity was measured by a dual-luciferase reporter gene assay system (Promega).

### Immunohistochemistry

Myocardium tissues were fixed in 10% Neutral buffer formalin and then embedded in OCT and cut into 6-μm-thick slices. After blocking with 3% hydrogen peroxide solution for 10 min, the sections were subsequently incubated overnight at 4 °C with the primary antibody (rabbit anti-mouse SIRT7, 1:200, ab78977, Abcam). Sections were then incubated with HRP-labeled goat anti-rabbit IgG (1:1000, Sigma) at 37 °C for 15 min. After three times of washing with PBS, the sections were stained with diaminobenzidine, and observed under an invert fluorescence microscope (Olympus Ckx53).

### Statistical analysis

All experiments were performed in triplicate and repeated at least three independent times. Data were presented as mean ± standard deviation (SD). Data were analyzed by the SPSS 22.0 statistical software (SPSS Inc., Chicago, IL) and GraphPad.Prism.v7.01. Student’s *t* test was used to compare the significant difference between two groups, and the One-way ANOVA test was applied when analyzing more than two groups. Tukey’s post hoc test was used to validate the ANOVA for comparing data between two groups. Differences were considered statistically at *P* < 0.05.

## Results

### Characterization of exosomes derived from BMSC

**Fig. 1:**
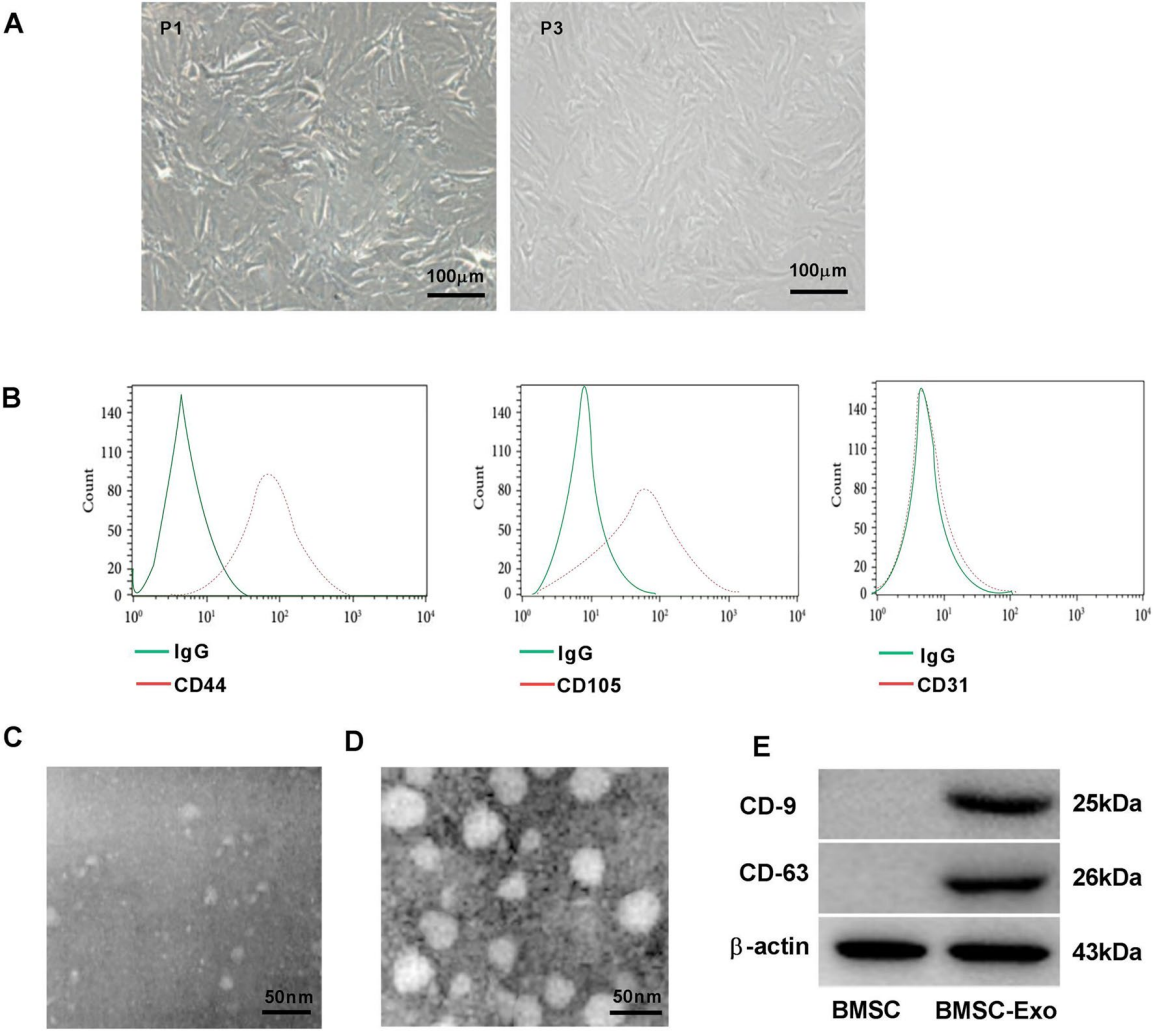
Characterization of exosomes derived from bone marrow mesenchymal stem cells (BMSCs). **a** Cellular morphology (P1, P3) of BMSCs observed under an inverted fluorescence microscope. Scale bar: 100 μm. **b** Flow cytometry was used to analyze the surface antigens (CD44, CD105, CD31) in BMSCs. **c**, **d** The morphology of FBS-derived exosomes and BMSC-derived exosomes was observed under transmission electron microscopy (TEM). Scale bar: 50 μm. **e** Western blot was used to examine the expression of CD9, CD63 in BMSCs, and BMSC-derived exosomes

As shown in Fig. 1a, BMSCs at first-passage (P1) were spindle-shaped, fusiform, and polygonal, and BMSCs at third-passage (P3) were spindle-shaped with stable morphology. The cells were identified as BMSCs on the basis of their spindle-shaped morphology, as well as their adherence to plastic. Flow cytometry analysis showed that BMSCs were positive for CD44, CD105, and negative for CD31 (Fig. 1b). In addition, we extracted exosomes from the supernatants of BMSCs. FBS-derived exosomes were not observed under TEM (Fig. 1c). Therefore, the interference of exosomes from FBS could be eliminated. Meanwhile, BMSC-derived exosomes were confirmed based on the round or oval shape, and 60–100 nm of diameter under TEM (Fig. 1d). Western blot confirmed the positive expression of characteristic cell surface antigens CD9 and CD63 in BMSC-derived exosomes. These results suggested that BMSC-derived exosomes were successfully extracted.

### Establishment of myocardial I/R model in rats

**Fig. 2:**
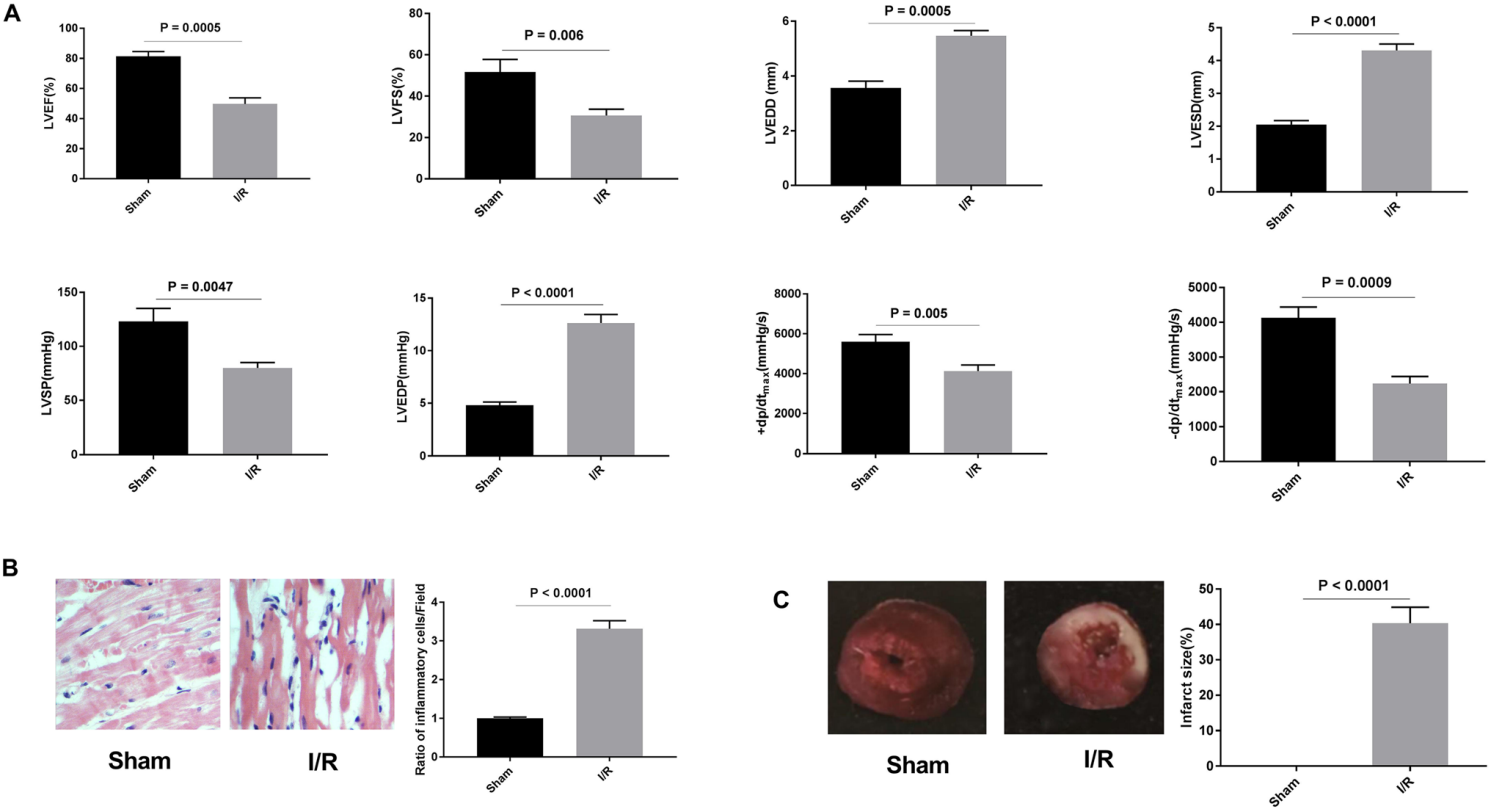
Myocardial ischemia reperfusion injury (I/R) model in rats. **a** Hemodynamic test of I/R rats. **b** Hematoxylin–eosin (HE) staining was performed to detect the pathological changes of myocardium in I/R rats (× 400). **c** Triphenyltetrazolium chloride (TTC) assay was used to evaluate the infarct size in I/R rats

A total of twenty rats were induced to myocardial I/R model. Among them, eighteen rats survived more than 2 weeks, and the survival rate was 90%. Cardiac function and hemodynamics of I/R rats were detected by TTE. The results showed that the LVEF, LVFS, LVSP, + d*p*/d*t*_max_, and − dp/d*t*_max_ were all reduced, and LVESD, LVEDD, and LVEDP were increased in I/R group, compared with Sham group (*P* < 0.01, Fig. 2a). HE staining suggested that myocardial fibers in Sham group were arranged neatly, and there was no inflammatory cell infiltration in stroma, while the myocardial fibers in I/R group were disorganized accompanied with infiltration of inflammatory cell and focal necrosis in the stroma (*P* < 0.0001, Fig. 2b). TTC assay was performed to detect the infarct size of myocardium tissues. Obvious myocardial infarction area was observed in I/R group, whereas no infarction area was observed in Sham group (*P* < 0.0001, Fig. 2c). All these data suggested that I/R model was successfully established in rats.

### BMSC-Exo-125b increased the expression of miR-125b in I/R myocardium cells

**Fig. 3:**
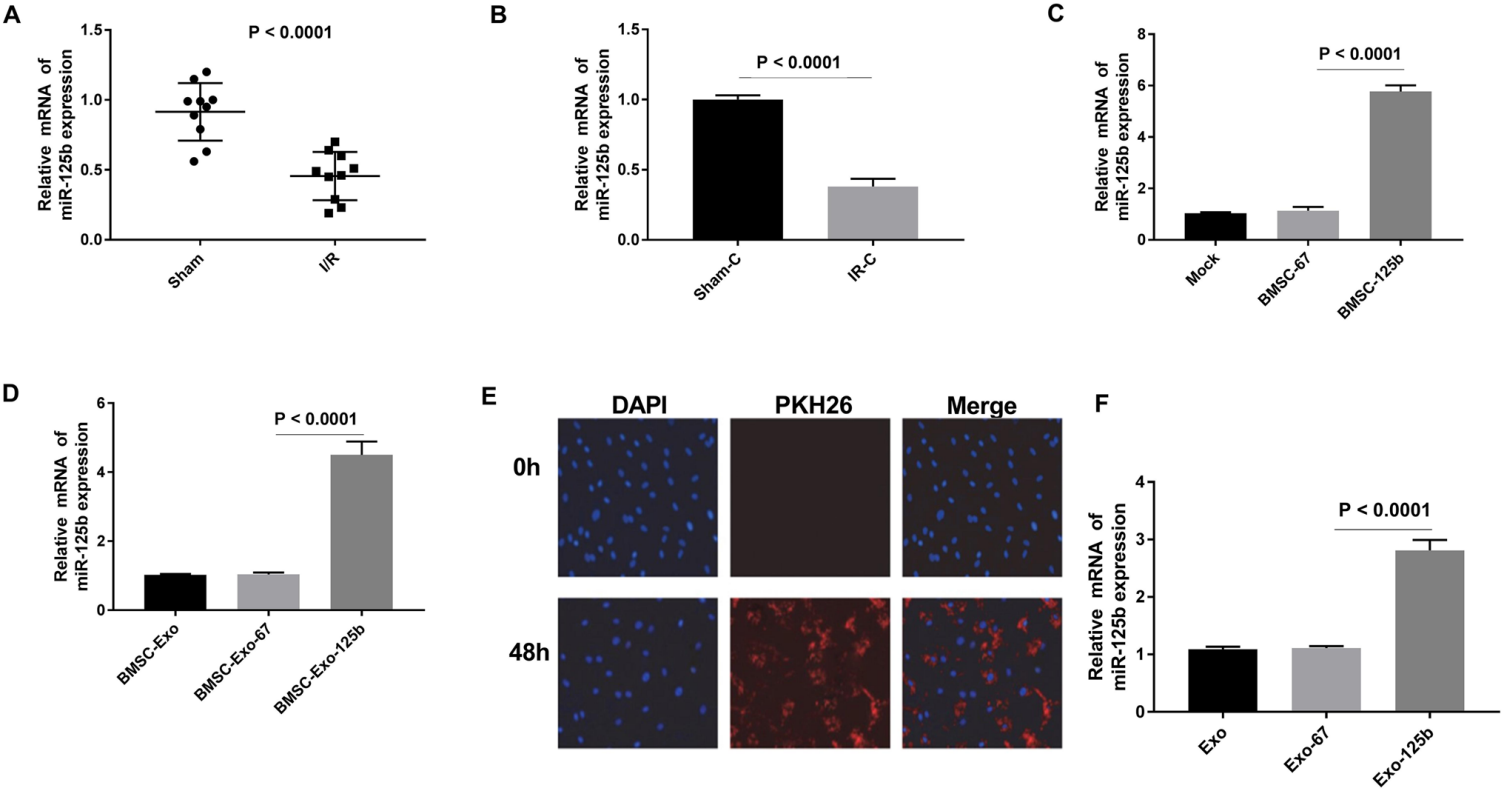
Bone marrow mesenchymal stem cells (BMSCs)-derived exosomes increased the expression of miR-125b in I/R myocardium cells. **a**, **b** The relative expression of miR-125b was examined by qRT-PCR in myocardium tissues and cells. **c**, **d** The relative expression of miR-125b was detected by qRT-PCR in BMSC cells and BMSC-derived exosomes, respectively. **e** BMSC-Exo-125b was traced by labeling PKH26 (red). **f** The relative expression of miR-125b was examined by qRT-PCR in I/R myocardium cells. (Color figure online)

To validate the effect of miR-125b on I/R, we measured the expression of miR-125b in myocardium tissues and cells. qRT-PCR showed that the expression of miR-125b in I/R group was markedly lower than that in Sham group (*P* < 0.0001, Fig. 3a, b). BMSC cells were transfected with Lv-miR-125b or cel-miR-67 (negative control) for 48 h. qRT-PCR showed that the expression of miR-125b was higher in miR-125b-transfected BMSCs than that in miR-67-transfected BMSCs (*P* < 0.0001, Fig. 3c). The expression of miR-125b was up-regulated in BMSC-Exo-125b-transfected cells, compared with BMSC-Exo-67-transfected cells (*P* < 0.0001, Fig. 3d). Meanwhile, BMSC-derived exosomes labeled with PKH26 (red) were traced. After transfection of Exo-125b, the relative miR-125b expression was increased in I/R myocardium cells (*P* < 0.0001, Fig. 3f). The above results suggested that BMSC-Exo-125b increased the expression of miR-125b in I/R myocardium cells.

### BMSC-Exo-125b enhanced the viability and inhibited the inflammation and apoptosis of I/R myocardium cells

**Fig. 4:**
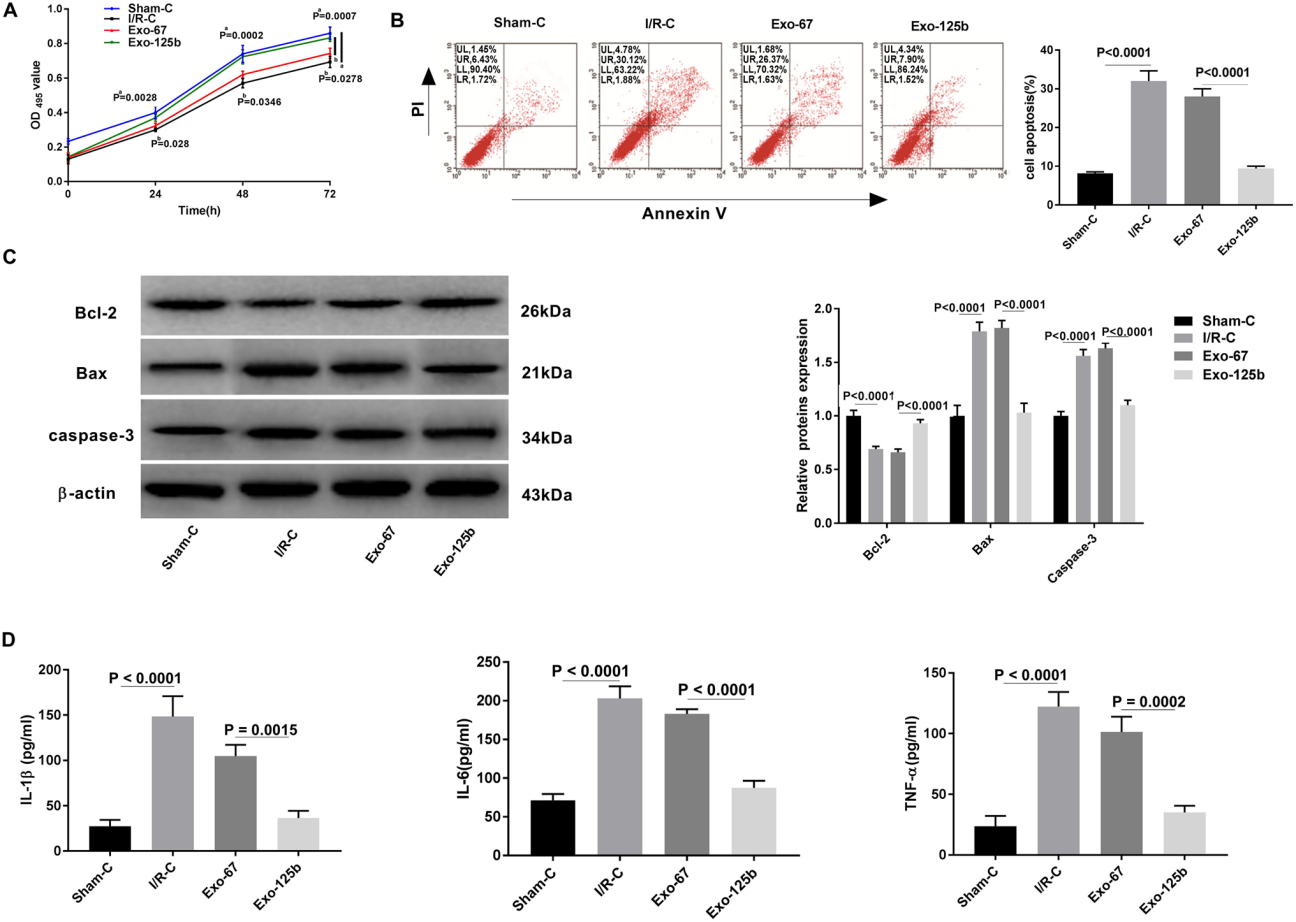
BMSC-Exo-125b inhibited the apoptosis of ischemia reperfusion injury (I/R) myocardium cells. **a** The viability of myocardium cells was examined by MTT. **b** Apoptotic ratio (%, cell ratio in LR + UR) of myocardium cells was examined by flow cytometry. Lower left quadrant (LL), viable cells; upper left quadrant (UL), necrotic cells; lower right quadrant (LR), early apoptotic cells; upper right quadrant (UR), late apoptotic cells. **c** Western blot was used to detect the expression of apoptotic proteins in myocardium cells. **d** ELISA was performed to examine the levels of inflammatory factors

The effect of BMSC-Exo-125b on the viability of I/R myocardium cells was analyzed by MTT assay. The result showed that the OD_495_ value was declined in I/R–C group compared with Sham-C group at 24, 48, and 72 h post-culturing (*P* < 0.01). The OD_495_ value of Exo-125b group was significantly higher than that of Exo-67 group at 24, 48, and 72 h post-culturing (*P* < 0.05, Fig. 4a). In contrast to OD_495_ value, the apoptotic ratio was significantly higher in I/R–C group than that in Sham-C group. The transfection of Exo-125b significantly decreased the apoptotic ratio compared with Exo-67 group (*P* < 0.0001, Fig. 4b). Western blot showed that the relative protein expression of pro-apoptotic factors Bax, caspase-3 was significantly higher in I/R–C group than in Sham-C group, and was significantly lower in Exo-125b group than in Exo-67 group (*P* < 0.0001). The Anti-apoptotic factor Bcl-2 exerted the opposite trend compared to the pro-apoptotic factors (Bax, caspase-3) (*P* < 0.0001, Fig. 4c). As presented in Fig. 4d, the levels of IL-1β, IL-6 and TNF-α were increased in IR–C group compared with Sham-C group (*P* < 0.0001). The transfection of Exo-125b significantly decreased the levels of IL-1β, IL-6 and TNF-α compared with Exo-67 group (*P* < 0.01, Fig. 4d). All these results demonstrated that Exo-125b enhanced the viability and inhibited the inflammation and apoptosis of I/R myocardium cells.

### MiR-125b was negatively correlated with SIRT7

**Fig. 5:**
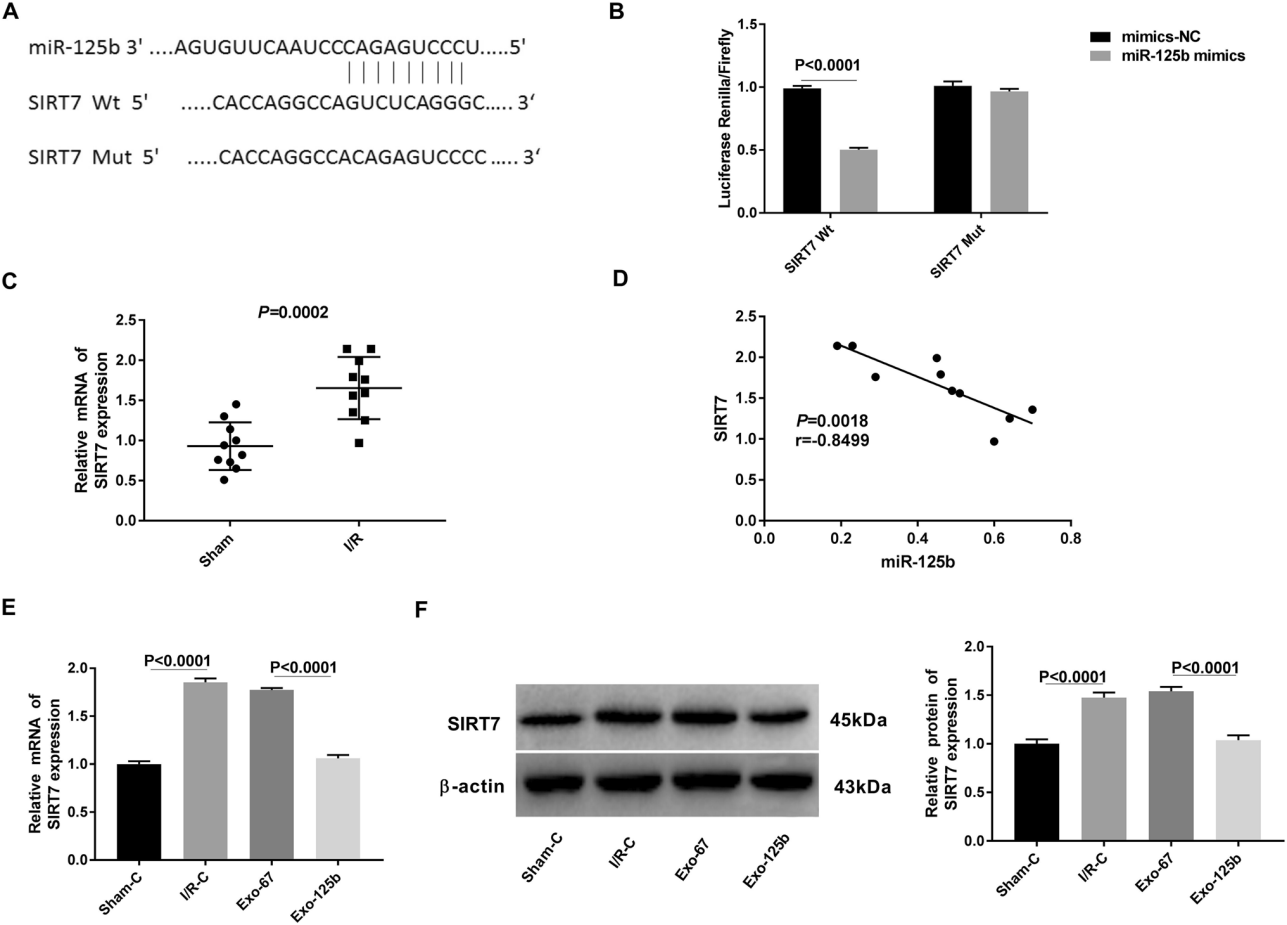
MiR-125b was negatively correlated with SIRT7. **a** A binding site at 3′-UTR of SIRT7 was predicted on miR-125b by StarBase3.0. **b** The luciferase activity was measured by dual-luciferase reporter gene assay. **c** Relative mRNA expression of SIRT7 in ischemia reperfusion injury (I/R) myocardium tissues was examined by qRT-PCR. **d** Spearman’s correlation analysis was used to detect the correlation between miR-125b and SIRT7 expression. **e**, **f** qRT-PCR and Western blot were used to detect the mRNA and protein expression of SIRT7 in myocardium cells

A binding site at 3′-UTR of SIRT7 was predicted on miR-125b by StarBase3.0 (Fig. 5a). Because SIRT7 plays an important role in the regulation of cell apoptosis and stress response in the heart [20–22], the relationship between SIRT7 and miR125b was further analyzed. Dual-luciferase reporter gene assay showed that the luciferase activity was decreased in myocardium cells co-transfected with SIRT7-Wt and miR-125b mimics, compared with that in cells co-transfected with SIRT7-Wt and mimics-NC (*P* < 0.0001, Fig. 5b). qRT-PCR showed that the expression of SIRT7 was significantly higher in I/R myocardium tissues (I/R group) than that in normal tissues (Sham group) (*P* = 0.0002, Fig. 5c). Spearman correlation analysis showed a negative relationship between SIRT7 and miR-125b in myocardium tissues (*r* = − 0.8499, *P* = 0.0018, Fig. 5d). In addition, the expression of SIRT7 in I/R myocardium cells (I/R–C group) was significantly higher than that in normal cells (Sham-C group) at both the mRNA and protein level (*P* < 0.0001). The transfection of Exo-125b significantly decreased the expression of SIRT7 in myocardium cells (Exo-125b group) compared with cells transfected with Exo-67 (Exo-67 group) at both the mRNA and protein level (*P* < 0.0001, Fig. 5e, f). Taken together, Exo-125b could negatively regulate the expression of its target gene SIRT7 in myocardium cells.

### BMSC-Exo-125b restored the cardiac function of I/R rats through down-regulating SIRT7

**Fig. 6:**
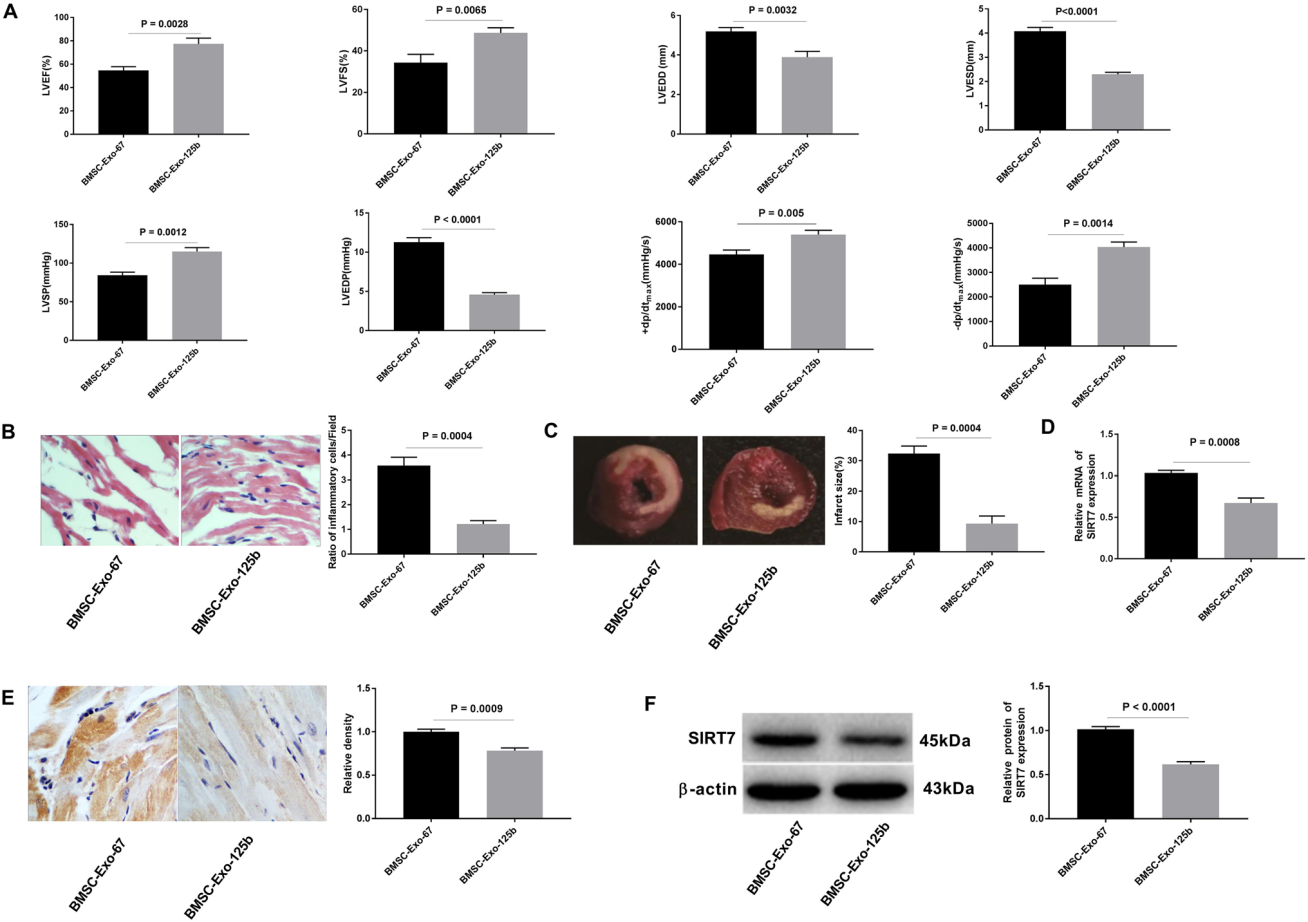
BMSC-Exo-125b restored the cardiac function of myocardial ischemia reperfusion injury (I/R) rats. **a** Hemodynamic test of I/R rats. **b** HE staining was performed to detect the pathological changes of myocardium in I/R rats (× 400). **c** Triphenyltetrazolium chloride (TTC) assay was used to evaluate the infarct size in I/R rats. **d** qRT-PCR was used to examine the relative mRNA expression of SIRT7 in myocardium tissues. **e** Relative density of SIRT7 was detected by immunohistochemistry in myocardium tissues (× 400). **f** Western blot was used to examine the relative protein expression of SIRT7 in myocardium tissues

To further evaluate the effect of BMSC-Exo-125b on cardiac function of I/R rats, BMSC-Exo-67 or BMSC-Exo-125b was injected into the left ventricular ligation area of rats, respectively. As shown in Fig. 6a, LVEF, LVFS, LVSP, + d*p*/d*t*_max_, and − dp/d*t*_max_ were significantly increased in I/R rats injected with BMSC-Exo-125b compared with those injected with BMSC-Exo-67 (*P* < 0.01). Meanwhile, LVESD, LVEDD, and LVEDP were decreased in I/R rats injected with BMSC-Exo-125b compared with those injected with BMSC-Exo-67 (*P* < 0.01). HE staining showed that ratio of inflammatory cells was reduced in BMSC-Exo-125b group compared with that in BMSC-Exo-67 group (*P* < 0.001, Fig. 6b). TTC staining showed that the infarct size was significantly lower in BMSC-Exo-125b group than that in BMSC-Exo-67 group (*P* = 0.0004, Fig. 6c). Furthermore, qRT-PCR, immunohistochemistry staining, and Western blot proved that the SIRT7 expression in BMSC-Exo-125b group was significantly lower than that in BMSC-Exo-67 group at both the mRNA and protein level (*P* < 0.001, Fig. 6e–f). To sum up, BMSC-Exo-125b could restore the cardiac function of I/R rats through down-regulating SIRT7.

## Discussion

Recently, I/R has become a research hotspot in cardiovascular pathology [23, 24] that is closely associated with vascular endothelial injury and myocardial cell apoptosis [25, 26]. In order to explore the treatment of myocardial I/R, we established I/R model in rats. I/R model is widely used in researches that focused on the biological function of various critical moleculars and signaling pathways. For example, Zhang et al. [27] verified that intravenous administration of NAD(+) can alleviate I/R through reducing apoptotic damage in a rat mode of myocardial I/R. In this study, LVEF, LVFS, LVSP, + d*p*/d*t*_max_, and − dp/d*t*_max_ were decreased, and LVESD, LVEDD, LVEDP were increased in I/R rats group. In addition, obvious myocardial infarction area was observed in I/R rats. These results demonstrate that the I/R model is successfully established in rats.

BMSCs attenuate hepatic I/R through inhibiting oxidative stress and suppressing apoptosis in rats [28]. Exosomes are regarded as small vesicles secreted by various types of cells, which play a critical role in paracrine effects. Exosomes derived from stem cells can ameliorate global function of the heart through preventing cell apoptosis, decreasing oxidative stress, and promoting angiogenesis under myocardial I/R [29–31]. Exosomes secreted by MSCs can reduce the infarct size in a mouse model of myocardial I/R [32]. In this study, exosomes were successfully extracted from the BMSCs, and used for subsequent assays.

Recently, accumulating evidence has proved that miRNAs participate in the regulation of myocardial cell apoptosis [33, 34]. Ma et al. [35] showed that the transplantation miR-132 exosomes in the ischemic hearts of mice markedly enhanced the neovascularization in the peri-infarct zone and preserved heart functions. The expression of miR-320 is remarkably down-regulated in I/R hearts, which can modulate the cardiac injury and dysfunction by negatively regulating Hsp20 [36]. Wang et al. [37] demonstrated that miR-494 exerts cardioprotective effects on I/R via targeting pro-apoptotic and anti-apoptotic proteins. In this study, our findings demonstrated that BMSC-Exo-125b enhanced the viability, and inhibited the apoptosis and inflammation of I/R myocardium cells. The inflammation and apoptosis of myocardium cells are key pathological characteristics of I/R [38]. Myocardial I/R promotes the formation of pro-inflammatory cytokines, and contributes to cardiac dysfunction and cardiomyocyte necrosis, as well as apoptosis [39]. The apoptotic signaling cascade can be initiated by free radicals and TNFα, both of which are produced in myocardial I/R [40]. Du et al. [41] proved that exendin-4, a glucagon-like peptide-1 receptor agonist attenuates myocardial I/R through inhibiting inflammation. Ma et al. [42] revealed that IL-33 protects against myocardial I/R by inhibiting inflammatory responses and myocardial apoptosis. Our results indicate that BMSC-Exo-125b inhibits the inflammation and apoptosis of myocardium cells, thereby contributing to the remission of I/R in myocardium.

SIRT7, which belongs to mammalian sirtuin family, plays an important role in oncogenic transformation [43]. Li et al. [44] proved that SIRT7 knockdown suppresses the proliferation and cell cycle progression of HUCCT1 cells (cholangiocarcinoma) in vitro and in vivo. Zhao et al. [45] demonstrated that miR-125b suppresses the proliferation of HepG2 cells (hepatocellular carcinoma) by targeting SIRT7. In this study, a binding site at 3′-UTR of SIRT7 was predicted on miR-125b. SIRT7 was further identified as a target of miR-125b. SIRT7 is involved in the regulation of cell apoptosis and stress response in the heart [20, 21]. For example, SIRT7 exerts a critical role in the regulation of stress response and cell death in the heart [20]. SIRT7 expression is increased in response to acute cardiovascular injury, including myocardial infarction and hind-limb ischemia, particularly at the active wound healing site [21]. Similarly with previous findings, we found that SIRT7 was up-regulated in I/R myocardium tissues and cells. We suspect that BMSC-Exo-125b may inhibit the inflammation and apoptosis of I/R myocardium cells through targeting SIRT7.

In conclusion, BMSC-Exo-125b enhanced the viability, inhibited the apoptosis and inflammation of I/R myocardium cells, and restored the cardiac function of I/R rats through regulating SIRT7. In other words, BMSC-derived exosomes carrying miR-125b protect against myocardial I/R by targeting SIRT7. BMSC-Exo-125b may serve as a potential therapeutic agent for myocardial I/R.

## References

[1] Eltzschig HK, Tobias E (2011). Ischemia and reperfusion–from mechanism to translation. Nat Med.

[2] Chen H, Xing B, Liu X, Zhan B, Zhou J, Zhu H, Chen Z (2010). Ischemic postconditioning inhibits apoptosis after renal ischemia/reperfusion injury in rat. Transpl Int Off J Eur Soc Organ Transpl.

[3] Hausenloy DJ, Yellon DM (2013). Myocardial ischemia-reperfusion injury: a neglected therapeutic target. J Clin Investig.

[4] Zhong-Yang Shen, Jing Zhang, Hong-Li Song, Wei-Ping Zheng (2013). Bone-marrow mesenchymal stem cells reduce rat intestinal ischemia-reperfusion injury, ZO-1 downregulation and tight junction disruption via a TNF-α-regulated mechanism. World J Gastroenterol.

[5] Haitao J, Linlin Q, Yun L, Lili G, Yichao S, Jian Z, Weiming Z, Jieshou L (2011). Bone marrow mesenchymal stem cells reduce intestinal ischemia/reperfusion injuries in rats. J Surg Res.

[6] Philippe M, Hagège AA, Jean-Thomas V, Michel D, Eric A, Bruno P, Alain B, Sorin S, Marcio S, Ketty S (2003). Autologous skeletal myoblast transplantation for severe postinfarction left ventricular dysfunction. J Am Coll Cardiol.

[7] Dohman HF, Perin E, Sousa A, Silva SA, Tinoco C, Esporcatte R, Rangel F, Campos LA, Fernandes MA, Dohmann H (2003). Transendocardial, autologous bone-marrow cell transplant in severe, chronic ischemic heart failure. Crit Care.

[8] Valadi H, Ekstrom K, Bossios A, Sjostrand M, Lee JJ, Lotvall JO (2007). Exosome-mediated transfer of mRNAs and microRNAs is a novel mechanism of genetic exchange between cells. Nat Cell Biol.

[9] Zhang W, Yang C, Guo W, Guo X, Bian J, Zhou Q, Chen M, Zhou J, Chen Z, Wang P (2018). Protective effect of bone marrow mesenchymal stem cells-derived exosomes against testicular ischemia-reperfusion injury in rats. J South Med Univ.

[10] Yan W, Zhao R, Liu D, Deng W, Xu G, Liu W, Rong J, Long X, Ge J, Bei S (2018). Exosomes derived from miR-214-enriched bone marrow-derived mesenchymal stem cells regulate oxidative damage in cardiac stem cells by targeting CaMKII. Oxid Med Cell Longev.

[11] Wang C, Zhu G, He W, Yin H, Lin F, Gou X, Li X (2019). BMSCs protect against renal ischemia-reperfusion injury by secreting exosomes loaded with miR-199a-5p that target BIP to inhibit endoplasmic reticulum stress at the very early reperfusion stages. FASEB J.

[12] Sheedy FJ, O’Neill LAJ (2008). Adding fuel to fire: microRNAs as a new class of mediators of inflammation. Ann Rheum Dis.

[13] Van-Rooij E, Marshall W, Olson EN (2008). Toward microRNA-based therapeutics for heart disease: the sense in antisense. Circ Res.

[14] Stephan F, Salvatore DR, Henrik F, Thomas S, Ariane F, Christoph L, Michael W, Hamm CW, Tino RX, Marga MA (2010). Circulating microRNAs in patients with coronary artery disease. Circ Res.

[15] Ye Y, Perezpolo JR, Qian J, Birnbaum Y (2011). The role of microRNA in modulating myocardial ischemia-reperfusion injury. Physiol Genomics.

[16] Liu LF, Liang Z, Lv ZR, Liu XH, Bai J, Chen J, Chen C, Wang Y (2012). MicroRNA-15a/b are up-regulated in response to myocardial ischemia/reperfusion injury. J Geriatr Cardiol.

[17] Xiaohui W, Tuanzhu H, Jianghuan Z, Danyang R, Li L, Xia Z, John K, Xiang G, David W, Chuanfu L (2014). MicroRNA-125b protects against myocardial ischaemia/reperfusion injury via targeting p53-mediated apoptotic signalling and TRAF6. Cardiovasc Res.

[18] Sheng CT, Ruenn Chai L, May May L, Choo ABH, Chuen Neng L, Sai Kiang L (2009). Mesenchymal stem cell secretes microparticles enriched in pre-microRNAs. Nucleic Acids Res.

[19] Zhao J, Li X, Hu J, Chen F, Qiao S, Sun X, Gao L, Xie J, Xu B (2019). Mesenchymal stromal cell-derived exosomes attenuate myocardial ischaemia-reperfusion injury through miR-182-regulated macrophage polarization. Cardiovasc Res.

[20] Vakhrusheva O, Smolka C, Gajawada P, Kostin S, Boettger T, Kubin T, Braun T, Bober E (2008). Sirt7 increases stress resistance of cardiomyocytes and prevents apoptosis and inflammatory cardiomyopathy in mice. Circ Res.

[21] Satoshi A, Yasuhiro I, Taku R, Alessandro I, Shinsuke H, Yuichi K, Yoshiro O, Takafumi S, Tatsuya Y, Osamu Y (2015). Sirt7 Contributes to myocardial tissue repair by maintaining transforming growth factor-β Signaling pathway. Circulation.

[22] Lv J, Tian J, Zheng G, Zhao J (2017). Sirtuin7 is involved in protecting neurons against oxygen-glucose deprivation and reoxygenation-induced injury through regulation of the p53 signaling pathway. J Biochem Mol Toxicol.

[23] Penna C, Brancaccio M, Tullio F, Rubinetto C, Perrelli MG, Angotti C, Pagliaro P, Tarone G (2014). Overexpression of the muscle-specific protein, melusin, protects from cardiac ischemia/reperfusion injury. Basic Res Cardiol.

[24] Riya G, Lytwyn MS, Pierce GN (2013). Differential effects of trans and polyunsaturated Fatty acids on ischemia/reperfusion injury and its associated cardiovascular disease States. Curr Pharm Des.

[25] Singhal AK, Symons JD, Boudina S, Jaishy B, Shiu YT (2010). Role of endothelial cells in myocardial ischemia-reperfusion injury. Vasc Dis Prev.

[26] Zhao D, Feng P, Sun Y, Qin Z, Zhang Z, Tan Y, Gao E, Lau WB, Ma X, Yang J (2018). Cardiac-derived CTRP9 protects against myocardial ischemia/reperfusion injury via calreticulin-dependent inhibition of apoptosis. Cell Death Dis.

[27] Zhang Y, Wang B, Fu X, Guan S, Han W, Zhang J, Gan Q, Fang W, Ying W, Qu X (2016). Exogenous NAD(+) administration significantly protects against myocardial ischemia/reperfusion injury in rat model. Am J Transl Res.

[28] Guangxin J, Gongcai Q, Dequan W, Yanhua H, Pengfei Q, Chengjuan F, Feng G (2013). Allogeneic bone marrow-derived mesenchymal stem cells attenuate hepatic ischemia-reperfusion injury by suppressing oxidative stress and inhibiting apoptosis in rats. Int J Mol Med.

[29] Xiao C, Wang K, Xu Y, Hu H, Zhang N, Wang Y, Zhong Z, Zhao J, Li Q, Zhu D (2018). Transplanted mesenchymal stem cells reduce autophagic flux in infarcted hearts via the exosomal transfer of mir-125b. Circ Res.

[30] Xiao J, Pan Y, Li XH, Yang XY, Feng YL, Tan HH, Jiang L, Feng J, Yu XY (2016). Cardiac progenitor cell-derived exosomes prevent cardiomyocytes apoptosis through exosomal miR-21 by targeting PDCD4. Cell Death Dis.

[31] Lucio B, Vincenzo L, Elisabetta C, Marco M, Mihaela G, Popescu LM, Tiziano T, Francesco S, Tiziano M, Giuseppe V (2017). Extracellular vesicles from human cardiac progenitor cells inhibit cardiomyocyte apoptosis and improve cardiac function after myocardial infarction. Cardiovasc Res.

[32] Lai RC, Arslan F, Lee MM, Sze NSK, Choo A, Chen TS, Salto-Tellez M, Timmers L, Lee CN, Oakley RME (2010). Exosome secreted by MSC reduces myocardial ischemia/reperfusion injury. Stem Cell Res.

[33] Chen CL, Yang J, James IO, Zhang HY, Besner GE (2014). Heparin-binding epidermal growth factor-like growth factor restores Wnt/β-catenin signaling in intestinal stem cells exposed to ischemia/reperfusion injury. Surgery.

[34] Zhang Z, Zhang H, Li H, Chen X, Liu M, Liu D, Zhao Y, Kong X (2014). Selective expression of tumor necrosis factor-related apoptosis-inducing ligand mediated by microRNA suppresses renal carcinoma growth. Mol Cell Biochem.

[35] Ma T, Chen Y, Meng Q, Sun J, Shao L, Yu Y, Huang H, Hu Y, Yang Z (2018). MicroRNA-132, Delivered by mesenchymal stem cell-derived exosomes, promote angiogenesis in myocardial infarction. Stem Cells Int.

[36] Xiao-Ping R, Jinghai W, Xiaohong W, Sartor MA, Jiang Q, Keith J, Persoulla N, Pritchard TJ, Guo-Chang F (2009). MicroRNA-320 is involved in the regulation of cardiac ischemia/reperfusion injury by targeting heat-shock protein 20. Circulation.

[37] Wang X, Zhang X, Ren XP, Chen J, Liu H, Yang J, Medvedovic M, Hu Z, Fan GC (2010). MicroRNA-494 targeting both proapoptotic and antiapoptotic proteins protects against ischemia/reperfusion-induced cardiac injury. Circulation.

[38] Mei Y, Jianchang C, Jing Z, Mei M, John C (2014). Etanercept attenuates myocardial ischemia/reperfusion injury by decreasing inflammation and oxidative stress. Plos ONE.

[39] Bonvini FR (2005). Inflammatory response post-myocardial infarction and reperfusion: a new therapeutic target?. Eur Heart J Suppl.

[40] Baines CP, Molkentin JD (2005). STRESS signaling pathways that modulate cardiac myocyte apoptosis. J Mol Cell Cardiol.

[41] Du X, Hu X, Wei J (2014). Anti-inflammatory effect of exendin-4 postconditioning during myocardial ischemia and reperfusion. Mol Biol Rep.

[42] Ma R, Hu X, Hu G, Yi C, Zhang C, Li X, Li Y, Jiang H (2015). Meijing W The protective role of interleukin-33 in myocardial ischemia and reperfusion is associated with decreased HMGB1 expression and up-regulation of the P38 MAPK signaling pathway. PLoS ONE.

[43] Ethan F, Renate V, Gregory L, Cornelia M, Ingrid G, Leonard G (2006). Mammalian Sir2 homolog SIRT7 is an activator of RNA polymerase I transcription. Genes Dev.

[44] Li W, Sun Z, Chen C, Wang L, Tao J (2018). Sirtuin7 has an oncogenic potential via promoting the growth of cholangiocarcinoma cells. Biomed Pharmacother.

[45] Zhao L, Wang W (2015). miR-125b suppresses the proliferation of hepatocellular carcinoma cells by targeting Sirtuin7. Int J Clin Exp Med.

